# Biological characteristics and pathogenicity of *Acanthamoeba*

**DOI:** 10.3389/fmicb.2023.1147077

**Published:** 2023-04-05

**Authors:** Yuehua Wang, Linzhe Jiang, Yitong Zhao, Xiaohong Ju, Le Wang, Liang Jin, Ryan D. Fine, Mingguang Li

**Affiliations:** ^1^College of Laboratory Medicine, Jilin Medical University, Jilin City, China; ^2^General Surgery, Jilin People’s Hospital, Jilin City, China; ^3^Department of Laboratory Medicine, Jilin Hospital of Integrated Chinese and Western Medicine, Jilin City, China; ^4^Center for Human Genetics and Genomics, New York University Grossman School of Medicine, New York City, NY, United States

**Keywords:** *Acanthamoeba*, biological characteristics, classification, disease, pathogenesis

## Abstract

*Acanthamoeba* is an opportunistic protozoa, which exists widely in nature and is mainly distributed in soil and water. *Acanthamoeba* usually exists in two forms, trophozoites and cysts. The trophozoite stage is one of growth and reproduction while the cyst stage is characterized by cellular quiescence, commonly resulting in human infection, and the lack of effective monotherapy after initial infection leads to chronic disease. *Acanthamoeba* can infect several human body tissues such as the skin, cornea, conjunctiva, respiratory tract, and reproductive tract, especially when the tissue barriers are damaged. Furthermore, serious infections can cause *Acanthamoeba* keratitis, granulomatous amoebic encephalitis, skin, and lung infections. With an increasing number of *Acanthamoeba* infections in recent years, the pathogenicity of *Acanthamoeba* is becoming more relevant to mainstream clinical care. This review article will describe the etiological characteristics of *Acanthamoeba* infection in detail from the aspects of biological characteristic, classification, disease, and pathogenic mechanism in order to provide scientific basis for the diagnosis, treatment, and prevention of *Acanthamoeba* infection.

## 1. Introduction

**FIGURE 1:**
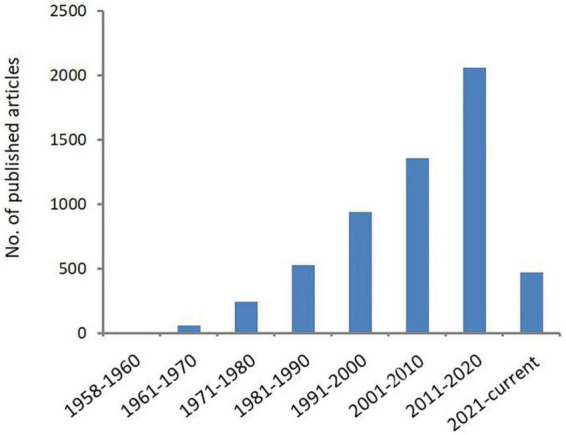
Number of publications related to *Acanthamoeba*. Increasing scientific interest in the field of *Acanthamoeba* as determined by published articles cataloged in PubMed over time.

*Acanthamoeba* is an opportunistic protozoa that is widely distributed in the natural environment, such as sea water, swimming pools, tap water, natural thermal water, soil, dust, and even the nasal mucosa of healthy individuals (Rivera et al., 1989, 1991; Michel et al., 1994; Tawfeek et al., 2016; Lass et al., 2017; Carnt et al., 2020; Wopereis et al., 2020). Pathogenic species can cause serious blindness in humans, arising from *Acanthamoeba* keratitis (AK) and rare granulomatous amoebic encephalitis (GAE) as well as skin and lung infections. Cases of AK and GAE were first reported by Fowler and Carter (1965) and by Naginton et al. (1974), respectively. The epidemiological investigation shows that the number of *Acanthamoeba* infection is increasing year by year, especially those with AK infection. In the United States, a study examining AK at thirteen ophthalmology centers and laboratories found that over the course of 2004–2007, a precipitous increase in AK cases had occurred: 22 cases were diagnosed in 1999, 43 cases were diagnosed in 2003, and 170 cases were diagnosed in 2007 (Yoder et al., 2012). In the UK, the numbers of AK cases diagnosed annually at the Moorfields Eye Hospital in London from 2011 to 2014 (range of 36–65 cases/year) were approximately two-to-three times higher than in 2004–2010 (range of 15–23 cases per year) (Carnt et al., 2018). In Australia, a study from the quaternary referral center in Sydney found that over the period from 2002 to 2016, the average annual number of cases was 50% higher post-2007 relative to pre-2007, and the highest numbers of cases were reported in 2007 and 2014 (Höllhumer et al., 2020). Studies found that wearing corneal contact lenses is the main risk factor for infection (Lindsay et al., 2007; Carnt and Stapleton, 2016; Dos Santos et al., 2018). With the increase of *Acanthamoeba* infection especially AK infection in recent years, scholars have begun expending extensive effort in characterizing and classifying the *Acanthamoeba* genus, as shown in Figure 1. This article summarizes the breadth of this work in order to provide scientific basis for the diagnosis, treatment, and prevention of *Acanthamoeba* infection.

## 2. Biological characteristics

### 2.1. Life history of *Acanthamoeba*

In general, the life cycle of *Acanthamoeba* consists of two stages, trophozoite and cyst (Marciano-Cabral and Cabral, 2003; Siddiqui and Khan, 2012; Siddiqui et al., 2012), except for *Acanthamoeba* pyriformis, which is recognized to include facultative sporocarp fruiting in its life cycle (Tice et al., 2016). The trophozoite stage dominates when the growth conditions are suitable, such as abundant food supply, neutral pH, appropriate temperature (i.e., 30°C) and osmolarity between 50 and 80 mOsmol (Siddiqui et al., 2012). The cell cycle of *Acanthamoeba* consists of growth and replication, which is carried out through mitosis under optimal living conditions (Band et al., 1970; Byers et al., 1991; Quinet et al., 2020). In axenic cultures, *Acanthamoeba* exhibits a typical exponential growth phase, followed by a period of reduced growth rate, and finally stationary phase during which no further increase in cell density occurs (Band and Mohrlok, 1973; Stevens and Pachler, 1973). Studies have revealed that the majority of *Acanthamoeba* cells in stationary phase culture develop into quiescent cysts in response to adverse conditions such as lack of food, hyper- or hypo-osmolarity, extremes in temperature and pH, high cell densities, and chemicals (Neff and Neff, 1969; Band and Mohrlok, 1973; Weisman, 1976; Stohr et al., 1987; Sriram et al., 2008). The trophozoites of *Acanthamoeba* mainly feed on bacteria, algae, yeast, or small organic particles *via* phagocytosis or pinocytosis and form many food vacuoles in the cytoplasm (Bowers, 1977; Bowers and Olszewski, 1983; Marciano-Cabral and Cabral, 2003). Pinocytosis is considered to be non-specific endocytosis (Bowers and Olszewski, 1972), while phagocytosis is considered to be receptor-dependent endocytosis (Alsam et al., 2005a). Once *Acanthamoeba* cells enter stationary phase, phagocytic activity ceases, while pinocytic activity is halved. The reduced pinocytic activity remains sensitive to respiratory inhibitors. The unequal responses of phagocytosis and pinocytosis to the onset of stationary-phase growth suggest that they are independent processes subject to different controls (Chambers and Thompson, 1976).

During the stationary phase shift, cysts begin to form with minimal metabolic activity (Siddiqui and Khan, 2012). Early in encystation, large numbers of vacuoles are observed in the encysting cells and many lysosomes and peroxisomes are present in the mature cysts (Müller, 1969; Müller and Moller, 1969; Siddiqui et al., 2012). Bowers and Olszewski (1983) showed that *Acanthamoeba* have the ability to distinguish vacuoles containing digestible and indigestible particles. For example, when cells were allowed to phagocytose yeast to capacity, endocytosis stopped and subsequent presentation of particles (either yeast or beads) did not result in further uptake. By contrast, when cells were allowed to phagocytose plastic beads to capacity and a second dose of particles was presented (either yeast or beads), the cells exocytosed the internal particles and took up the new ones. Therefore, the fate of vacuoles containing yeast and vacuoles containing plastic beads in encystation is different (Bowers and Olszewski, 1983). The digestive vacuoles disappear during later stages of encystation and their contents are discharged. This phenomenon explains the weakening of phagocytic activity and pinocytic activity in this period. Cysts are highly tolerant to the extreme environmental conditions, which allows *Acanthamoeba* to spread in the environment and/or carry these pathogens into host species, which is described later in the text.

### 2.2. The morphological structure of *Acanthamoeba*

#### 2.2.1. Trophozoite

**FIGURE 2:**
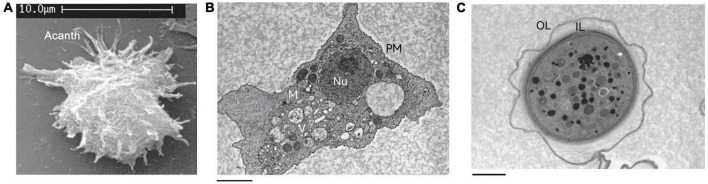
The structure of *Acanthamoeba*. **(A)** Scanning electron micrograph of an *Acanthamoeba* trophozoite showing many spinous pseudopods around the whole surface of the cell. Acanth, acanthopod. **(B)** Transmission electron micrograph of the trophozoite stage of *Acanthamoeba*. Nu, nucleus; V, vacuoles; M, mitochondria; PM, plasma membrane. **(C)** Transmission electron micrograph of an *Acanthamoeba* cyst. OL, outer layer; IL, inner layer. These pictures are from the work of Siddiqui et al. (2012).

The trophozoite varies in size from 25 to 40 μm in diameter (Marciano-Cabral and Cabral, 2003) and has a long oval or irregular shape. The cytoplasmic boundary of trophozoite is unclear and the endoplasm is granular, which emits several acicular or spinous pseudopods extending to the whole surface of the cell (Figure 2A). The pseudopods give the trophozoite a characteristic appearance and participate in the feeding and movement of *Acanthamoeba*. Pseudopods in *Acanthamoeba* have a peculiar form. The term “acanth” is a Greek word meaning “spike” to indicate the presence of spine-like structures (also known as acanthopods) on the surface of the amoeba (Preston and King, 1984; Preston et al., 2001; Marciano-Cabral and Cabral, 2003). Pseudopods are responsible for adhesion to the surface of contact lenses, increasing the chance of *Acanthamoeba* infection in the cornea (John et al., 1989; Omaña-Molina et al., 2014). The trophozoite is typically uninucleate with a nucleus that is approximately one sixth the size of the cell body, but multinucleated individuals can be seen in laboratory cultures when grown with constant agitation (Byers and James, 1967). To some extent, this phenomenon reflects the real living condition of *Acanthamoeba* in nature because the existence of *Acanthamoeba* can often be found in an aquatic environment. Any flux perturbations, such as water currents or waves, might detach amoebae from their substrate and suspend them in the water in a non-adherent state, which creates conditions for the formation of multinucleation. This process will help amoebae to colonize new environmental niches that are difficult to reach using an adherent state. When adhesion is restored, multinucleated amoebae generate a higher progeny population compared to uninucleate mother cells. This process is beneficial from the point of view of continual population reproduction (Quinet et al., 2020). The nuclear envelope is separated by a distance of about 350 Å with numerous nuclear pores (Bowers and Korn, 1968). Chromatin is located on the inner surface of the nuclear envelope. The nucleolus is perhaps the most striking feature appearing large and dense and surrounded by a unique zona pellucida (Pussard and Pons, 1977). In the trophozoite stage, *Acanthamoeba* does not differ greatly at the internal structural level from a mammalian cell. It also contains various cell organelles such as mitochondria, ribosomes, centrosome, Golgi apparatus, and vacuoles (Bowers and Korn, 1968; Figure 2B). Vacuoles are conspicuous elements in trophozoite. They exist mainly in two separate systems: one is the contractile vacuole, involved in cellular osmotic regulation (Kitching, 1967) and the other is the digestive vacuole involved in the decomposition of intake particles (Kitching, 1956). Contractile vacuoles are periodically expelled and then refilled in a specific manner, ranging in size from 0.1 μm in diameter to larger than the nucleus. Multiple small vacuoles fuse together to form large vacuoles, which are morphologically akin to digestive vacuoles (Bowers and Korn, 1968). The contractile vacuole can be distinguished from a digestive vacuole by the absence of flocculent content when examined by electron microscopy (Bowers and Korn, 1968). In addition to contractile vacuoles and digestive vacuoles, other types of vacuoles can also be observed in the cytoplasm such as lysosomes and a large number of glycogen-containing vacuoles (Bowers and Korn, 1973; Siddiqui and Khan, 2012).

#### 2.2.2. Cyst

There can be a lot of variability in cyst shape, most cysts are round and the diameter is about 13–20 μm (Bowers and Korn, 1969). Unlike trophozoites, the cyst has a double-layer cell wall, which gives cysts strong resistance to many harsh environmental conditions (Figure 2C). The outer layer consists of a laminar fibrous layer also known as an ectocyst, while the inner layer is made up of fine fibers also known as an endocyst. The ectocyst is the first to be synthesized in the encysting cells and appears as an amorphous discontinuous layer just outside the plasma membrane (Bowers and Korn, 1969). The ectocyst terminates in a loose fibrous layer and the entire ectocyst is 0.3–0.5 μm in thickness. The endocyst differs from the ectocyst in texture and appears finely granular. The fibrils of both the ectocyst and the endocyst appear to be less than 50 Å in diameter. When the cysts are fully mature, a space of about 0.1 μm is formed between the endocyst and the plasma membrane (Bowers and Korn, 1969).

The cyst wall of cyst forming protozoa is generally composed of chitin, while *Acanthamoeba* is an exception and includes both chitin and cellulose (Linder et al., 2002; Derda et al., 2009). Cellulose becomes the main component of the endocyst, accounting for about one third of the cyst wall (Tomlinson and Jones, 1962). However, it is not clear where chitin exists in ectocyst or endocyst. Cellulose is basically a straight chain polymer of β-1, 4 linked D-glucose units which are arranged in alternate orientation with respect to one another (Béguin and Aubert, 1994; Lakhundi et al., 2015). It is with no coiling and rod-like conformation that provokes spontaneous crystallization of the molecule (Schwarz, 2001). An exceptional feature of cellulose that is also relatively unusual in the polysaccharide world is its crystalline structure (Brown and Saxena, 2000; Lynd et al., 2002). Approximately 30 individual molecules of cellulose are assembled into larger units called elementary fibrils (protofibrils), which in turn are packed into larger units called microfibrils (Brown and Saxena, 2000; Lynd et al., 2002). The chains in the microfibrils are held together by hydrogen bonds giving them a high tensile strength. It is this inter- and intrachain hydrogen bonding between multiple parallel layers of cellulose that results in the formation of tightly packed microfibrils. The microfibrils then associate into crystalline cellulose fibers. The organization of individual microfibrils in crystalline cellulose is such that the component molecules are packed tightly enough to prevent penetration by enzymes. In addition, cellulose also contain various irregularities such as twists or voids, surface micropores, large pits and capillaries etc., increasing the total surface area much larger than that of an ideally smooth fiber of the same dimensions (Blouin et al., 1970; Cowling, 1975; Fan et al., 1980; Lynd et al., 2002). Overall, cellulose imparts high tensile strength to the wall it is contained in, serving as a structural component. It is an extracellular polysaccharide and is a part of the cell wall in plants, algae, bacteria, slime mold Dictyostelium and other protists such as the *Acanthamoeba* cyst wall (Schramm and Hestrin, 1954; Cook and Colvin, 1980; Williams and Cannon, 1989; Bishop et al., 2002). Given the structure of cellulose, it practically makes it impossible for an enzyme to clasp cellulose into a substrate site. Hence there is only a single enzyme to hydrolyze cellulose. This, together with its association with other polymers, makes cellulose containing material withstand harsh conditions making it hardy and resistant to degradation, hence its role as a structural and protective barrier.

The change of environmental factors is an important link in the transformation of trophozoites into cysts. Cyst formation occurs under adverse environmental conditions such as glucose deficiency, dryness, extreme temperature, and non-neutral pH environments (Bowers and Korn, 1969; Chagla and Griffiths, 1974; Byers et al., 1980). Even more notable is the durability of cysts with one study noting that cysts can grow after 21 years even in a completely dry environment while another conversely showed cysts surviving in water at 4^°^C for more than 24 years (Mazur et al., 1995; Sriram et al., 2008). Cysts have been shown to be resistant to fungicides, chlorinating agents, and antibiotics (De Jonckheere and van de Voorde, 1976; Khunkitti et al., 1998; Turner et al., 2000; Lloyd et al., 2001). However, chloroquine effectively inactivated the cells in a dose-dependent manner during encystation (Jha et al., 2014). This result indicates that there is a potential target of chloroquine in the process of encystation, while also suggesting the importance of timing for the treatment of *Acanthamoeba* infection.

The viability and virulence of 17 *Acanthamoeba* isolates after they have been stored in water at 4°C for a period of 24 years was determined, and 3 of them were found inactivated. The remaining 14 isolates after inoculation on non-nutrient agar (NNA) gave rise to new subculture. Due to the low number of viable cysts (0–5%) measured by eosin exclusion, most of the cells entered the trophozoite stage. These results revealed the resistance and viability of *Acanthamoeba* in long term storage (Mazur et al., 1995). Separate groups of mice were inoculated intranasally with 10 subcultures characterized by varying primary virulence. In 8 groups, the mice were successfully infectious (at varying degree), and some of the mice succumbed to disease, indicating that even after 24 years, some of strains still keep very high virulence. Interestingly, however, some of the examined isolates completely lost their virulence only after 8 years of cultivation. On the basis of these results, we can assume in the environment that the period of viability for a cyst may be at least 25 years, and, more importantly, some of them still maintain high virulence (Mazur et al., 1995). It is this durability of cysts to the external environment and drugs that makes the treatment of *Acanthamoeba* infection particularly difficult. However, a 0.02% polyhexamethyl biguanide (PHMB) solution can be used as a first line of defense topical therapy for AK (Schuster and Visvesvara, 2004; Oldenburg et al., 2011; Carrijo-Carvalho et al., 2017) and is recommended by the leading national public health organizations in the USA (Centers for Disease Control and Prevention, 2020). A recent phase I study of human subjects has shown general tolerance to higher concentrations of PHMB, which makes it possible to treat deep stromal invasion of AK (Papa et al., 2022). PHMB 0.08% monotherapy is being further explored in a phase III randomized control trial for AK (ClinicalTrials.gov/ NCT03274895, National Library of Medicine, 2020).

#### 2.2.3. Encystation

##### 2.2.3.1. Cellulose and encystation

Encystation is an essential biological process for survival of cyst forming protozoa (Eichinger, 2001). In the process, *Acanthamoeba* undergoes drastic changes in gene expression to adapt to the new environment (Moon et al., 2009b,2011b; Bernard et al., 2022). The most prominent characterization is the formation of cyst wall, which serves as a shelter under stressful external conditions (Marciano-Cabral and Cabral, 2003; Turner et al., 2004). The cyst wall contains at least 2 major products that are not detected in the trophozoite stage; cellulose (Tomlinson and Jones, 1962) and an acid-insoluble protein-containing material (Neff and Neff, 1969).

As described above, cellulose constitutes a major component of the *Acanthamoeba* defense system during encystation. The precursor of cellulose is glucose that is incorporated into the cell wall as β (1, 4)-glucans (Weisman, 1976). *Acanthamoeba* stores glucose in the form of glycogen in the active growth stage and degrades it to form glucose during the process of encystation before final conversion into cellulose (Weisman et al., 1970; Potter and Weisman, 1971; Stewart and Weisman, 1974). It has been demonstrated that glycogen is the most rapidly degraded macromolecule during the initial phase of *Acanthamoeba* encystation (Bowers and Korn, 1969; Weisman et al., 1970). Glycogen is broken down into glucose *via* glycogen phosphorylase (Lorenzo-Morales et al., 2008; Moon and Kong, 2012). This was demonstrated by gene silencing of glycogen phosphorylase, which prevented *Acanthamoeba* from forming a double-layered cyst wall (Lorenzo-Morales et al., 2008). There has been some debate about cellulose existing only in the endocyst (Tomlinson and Jones, 1962). However, a recent study by Garajová et al. (2019) revealed cellulose fibers through several electron microscopy methods and observed cellulose in the outer layer. *Acanthamoeba* transfected with siRNA of glycogen phosphorylase can still form outer layer of cyst wall during encystation, which may be due to the differences in the mechanisms that provide glucose for the synthesis of polysaccharides in the inner and outer cyst wall layers. It may also be attributed to the fact that the lack of cellulose is not enough to hinder the formation of the basic structure of outer layer because the main components of the ectocyst are proteins (Barrett and Alexander, 1977; Dudley et al., 2009). However, no matter what, the fact that glycogen phosphatase is required for cyst wall assembly, mainly the cell wall inner layer, has been demonstrated (Lorenzo-Morales et al., 2008). The gene expression level of glycogen phosphorylase also gradually increases during cyst formation and has been shown to reach a maximum at day 3 (10.3 times) (Moon and Kong, 2012). After glycogen breakdown, glucose is then converted to cellulose by the enzyme cellulose synthase (Delmer and Amor, 1995). Targeting cellulose synthase by siRNA has been shown to significantly inhibit the formation of mature cysts (Aqeel et al., 2013; Moon et al., 2014). The sturdy nature of *Acanthamoeba* cysts is attributed, in part, to cellulose (Potter and Weisman, 1972), but other polysaccharides (such as xylose, galactose, and mannose) may also be involved (Dudley et al., 2009). The analysis of cyst walls of *Acanthamoeba castellanii*, using gas chromatography/mass spectrometry (GC/MS) revealed that xylose (in addition to β-1, 4-glucan-forming cellulose) was another important component of the cyst wall (Dudley et al., 2009). Xylose isomerase is involved in the intracellular transformation of xylose. Xylose isomerase, also known as glucose isomerase, catalyzes the reversible isomerization of xylose into xylulose and glucose into fructose (Lee et al., 1990). An siRNA against xylose isomerase and its exogenous inhibitor, sorbitol, blocked *A. castellanii* encystation, suggesting that xylose isomerase plays an important role in amoebae differentiation (Aqeel et al., 2013). Aqeel et al. (2013) proposed that xylose biosynthesis could replace cellulose biosynthesis, resulting in hemicellulose entering the cyst wall and forming immature cysts. However, Moon et al. (2014) showed that cellulose synthetase and xylose isomerase were expressed independently in encysting *Acanthamoeba*, and that one cannot replace each other. Therefore, how xylose isomerase interferes with cyst wall formation leading to reduced encystation in *A. castellanii* needs to be further studied.

##### 2.2.3.2. Autophagy and encystation

Autophagy is a degradative pathway necessary for the clearance of damaged or superfluous proteins and organelles and the recycling of intracellular constituents as well as providing energy during periods of unfavorable environments such as under nutrient-limiting conditions (Huang and Klionsky, 2002; Xie and Klionsky, 2007). Autophagy starts with the formation of a double-layered membrane, called a phagophore, which recruits proteins and lipids into a presumed membrane structure known as the pre-autophagosomal structure (PAS) through a series of reactions involving autophagy (Atg) proteins (Kim et al., 2002; Suzuki et al., 2002). The phagophore is enlarged and then forms an autophagosome that encloses the cytosolic components and organelles including mitochondria and endosomes (Ishihara et al., 2001; Reggiori et al., 2005). Thereafter, mature autophagosomes fuse with lysosomes and their contents are degraded (Nakatogawa, 2020).

The formation of autophagosome involves two ubiquitin like conjugation systems (UBL); Atg8 and Atg12 systems (Ohsumi and Mizushima, 2004). In the Atg8 conjugation system, Atg8 is cleaved at the C-terminal end by a cysteine protease, Atg4, producing Atg8*^Gly–116^* (Kirisako et al., 2000). It is then transferred to Atg3, an E2-like enzyme, after being activated by Atg7, an E1-like enzyme (Ichimura et al., 2000, 2004). Finally, Atg3 conjugates Atg8 with phosphatidylethanolamine (PE) (Ichimura et al., 2000), anchoring Atg8 to the autophagosome membrane. In the Atg12 conjugation system, the C-terminal glycine residue of Atg12 is activated by Atg7, an E1-like enzyme, transferred to Atg10, an E2-like enzyme, and conjugated to Atg5 covalently (Geng and Klionsky, 2008). The Atg12-Atg5 conjugate which has E3-like activity promotes the elongation of the autophagosomal membrane by forming a multimeric complex with Atg16 (Hanada et al., 2007; Yang and Klionsky, 2009). Later studies have found that Atg12-Atg5 conjugates directly activate E2 enzyme activity of Atg3 to promote conjugation of Atg8 to phosphatidylethanolamine (Sakoh-Nakatogawa et al., 2013), indicating that two UBL systems are not completely independent in mediating autophagy.

The Atg8 and Atg12 UBL systems are well conserved in *A. castellanii* (Moon et al., 2011a; Song et al., 2012). The mediation of Atg8 and Atg12 systems in the autophagy process during encystation has been identified and autophagy proteins including Atg3, Atg8, Atg8b (isoform of Atg8), Atg12 and Atg16 have been characterized (Moon et al., 2009a,2011a,^2013^; Song et al., 2012; Kim et al., 2015). The mRNA expression level of Atg8, Atg8b, and Atg16 are highly induced during encystation (Moon et al., 2009a,^2013^; Song et al., 2012). However, the transcriptional level of Atg3 and Atg12 did not change markedly in both trophozoites and cysts (Moon et al., 2011a; Kim et al., 2015). Therefore, it can be speculated that Atg3 and Atg12 proteins are used in the form of recycling during encystation. The discrepancy on gene expression does not cast their role in encystation into doubt since siRNA against the respective genes inhibited cyst formation effectively. Atg3, which is uniformly distributed in trophozoite cells, aggregates around the autophagosomal membrane in encystation and shows an activity for Atg8 lipidation (Moon et al., 2009a). As mentioned above, ATG8 is one of the ubiquitin-like proteins required for autophagosome formation. PE conjugated ATG8 is tightly bound to the autophagosome membrane and participates in autophagy (Moon et al., 2009a). Complete inhibition of encystation was not achieved in *Acanthamoeba* transfected with siRNA against Atg8, which suggests that an Atg8 isoform such as Atg8b exists (Moon et al., 2013). Both Atg8 and Atg8b were highly expressed during encystation, probably because they were needed to enable the establishment of autophagy rapidly. Atg12 together with Atg16 constituted another UBL system related to autophagy (Song et al., 2012) and the knockdown of Atg12 or Atg16 showed ultrastructural changes of the cyst. The main features of Atg12-knockdown cells are the absence of maturation of cyst wall, decrease in autophagic structures, and vacuolization (Kim et al., 2015). The ultrastructural characteristics of Atg16-knockdown cells showed that many mitochondria were still undigested and these cells are prevented from forming mature cysts, which supports the view that autophagy is necessary for the effective degradation of mitochondria during encystation in *Acanthamoeba* (Song et al., 2012).

Proteases from various protozoan parasites have been characterized at the molecular and cellular levels (Klemba and Goldberg, 2002). Comparative microarray analysis of trophozoite and cyst showed high expression of cysteine in the cyst stage (282-fold change) (Moon et al., 2011b), suggesting a pivotal role of this protease in the cyst formation. Because of its high expression in cysts, people named the gene cyst specific cysteine protease (CSCP). During encystation, CSCP showed colocalization with LysoTracker, an autophagosome marker. *Acanthamoeba* transfected with siRNA against CSCP was unable to form mature cysts and the undigested mitochondria in vacuole-like structures were observed in CSCP siRNA treated cells (Moon et al., 2012b). Mitochondria are a major target of autophagy in *Acanthamoeba*, as compared with trophozoites, the number of mitochondria left in mature cysts is significantly reduced (Moon et al., 2012b). To degrade a large number of mitochondria, various types of autophagy may be needed. As mentioned above, Atg16, as an important component of Atg 12 UBL system, together with CSCP involved in the degradation of mitochondria during encystation. Further studies need to clarify the possible interaction between these two proteins in this regulatory process.

The encystation of *Acanthamoeba* was inhibited by the serine protease inhibitor phenylmethanesulfonyl fluoride, indicating that serine protease was also involved in encystation (Moon et al., 2008b). To confirm the role of encystation-mediating serine protease (EMSP) during encystation of *Acanthamoeba*, a gene silencing assay was performed and showed that the formation of mature cysts was almost completely inhibited in EMSP siRNA-transfected cells. Additionally, both gene and protein expression of EMSP are highly induced during encystation (Moon et al., 2008b). However, the increased gene level of EMSP during encystation was not revealed by comparative microarray analysis of trophozoite and cyst (Moon et al., 2011b). This may be due to the inconsistency of sample collection timing in the process of encystation or the use of different strains, *A. healyi* instead of *A*. *castellanii*. The protein turnover during *Acanthamoeba* encystation has also been studied by two-dimensional gel electrophoresis (2DE). The results showed that protein degradation mainly occurred early in the process and these changes could be significantly inhibited specifically by cysteine protease inhibitors. The conclusion is that the encystation process in *A. castellanii* is of a bipartite nature consisting of an initial phase of protein degradation by a cysteine protease and the late stage accompanied by the expression of cyst-specific gene expression (Leitsch et al., 2010).

##### 2.2.3.3. Other factors of encystation

A cDNA fragment containing a member of the sirtuin family of proteins was found in a comparative microarray analysis of trophozoites and cysts (Moon et al., 2011b). Sirtuins are a silent-information regulator 2 (SIR2)-like family of protein deacetylases that require nicotinamide adenine dinucleotide (NAD^+^) as a cofactor in the deacetylation reaction (Sauve et al., 2001). The Sir2 homolog of *A. castellanii* (AcSir2) contains the YEATS domain which recognizes and binds acetylated lysine followed by a Sir2 catalytic domain. Nuclear extracts of AcSir2-overexpressing cells also exhibit NAD^+^ dependent deacetylase activity (Joo et al., 2020). The overexpression of AcSir2 converted cells into mature cysts more rapidly while the encystation of *A. castellanii* was suppressed by treatment with salermide, a sirtuin inhibitor. The transcription of cellulose synthase was induced in AcSir2 overexpressing cells while the transcription was completely abolished in salermide-treated cells (Joo et al., 2020), which indicated that cellulose synthesis may serve as a potential target of Sir2. The same group also found that AcSir2 promotes encystation by increasing the expression of cyst-specific cysteine protease (CSCP). Sirtinol, another Sir2 inhibitor, suppresses CSCP transcription, suggesting that the undegraded organelles and large molecules remained in sirtinol-treated cells during encystation (Joo et al., 2022). In *Saccharomyces cerevisiae*, SIR2 levels increase during calorie restriction (Lin et al., 2000), and sirtuin overexpression is known to extend the lifespan by silencing HML and HMR loci and inhibiting the formation of extrachromosomal rDNA circles (Sinclair and Guarente, 1997; Kaeberlein et al., 1999). Therefore, it was proposed that Sirtuins may be involved in all cell survival events related to metabolic reduction caused by nutritional deficiency, including *Acanthamoeba* encystation.

Bernard et al. (2022) studied the regulation of transcription, protein and phosphoprotein level in the early stage of *Acanthamoeba* encystation by a time-resolved multi-omics analysis. The global analysis of three omics approaches showed that the quantity of transcripts and phosphorylation sites were modified as early as 1 h after triggering encystation while the change of proteome was more gradual and occurred 8 h later. Interestingly, 1 h after induction of encystation, a decrease of phosphorylated sites was observed and accompanied by a global increased phosphatase activity (Bernard et al., 2022). *A. castellanii* is predicted to encode the largest number of protein kinases among amoebozoans (Clarke et al., 2013). However, only few signal pathways, such as PKC, Ras, and cyclic AMP (cAMP) have been proposed to be involved in encystation (Raizada and Krishna Murti, 1972; Fouque et al., 2012; Moon et al., 2012a), and there is no clear link between signals and cellular response. During encystation, these kinases may be involved in the regulation of phosphorylation at a specific site that is currently undefined. Bernard et al. (2022) also explained the transcriptomic and proteomic data at the initial stage of encystation. Their hypothesis is that the early regulation of transcription is achieved by the repression of transcription factors through phosphorylation. Some of these factors like GSK3 are highly promiscuous with a broad array of known substrates, regulate many transcription factors such as Fos/Jun AP-1 or p53 (Beurel et al., 2015). Lectins are an important part of cyst wall and three sets of these proteins have been identified as the most abundant in *A. castellanii*, which are named as the Jonah, Luke, and Leo families (Magistrado-Coxen et al., 2019). 23 out of 31 lectins described by Magistrado-Coxen et al. (2019) were present in transcriptomic data and 16 were significantly differentially expressed. However, none of them were significantly regulated at the protein level. This may be due to timing because their expression peak may be later than 8 h after induction of encystation (Magistrado-Coxen et al., 2019; Bernard et al., 2022). Part of the transcriptomic data in the multi-omics analysis (Bernard et al., 2022) may come from epigenetic regulation in the early stage of encystation. Epigenetics is associated to many cellular processes such as gene and microRNA expression, DNA-protein interactions, suppression of transposable element mobility, cellular differentiation, embryogenesis, etc. (Portela and Esteller, 2010). *A. castellanii* transfected with siRNA against Protein Arginine Methyltransferase 5 (AcPRMT5) failed to form mature cysts (Moon et al., 2016). DNA methylation is also involved in the control of CSCP expression during encystation (Moon et al., 2017).

In addition, several genes related to *Acanthamoeba* encystation have been identified from encystation-related gene profiles, such as cyst specific protein 21, Na P-type ATPase, subtilisin-like serine proteinase genes, proteasome and heat shock protein, genes like cullin 4, ubiquitin-conjugating enzymes, suggesting their potential roles in the process of cyst formation (Moon et al., 2007, 2008a).

### 2.3. Classification of *Acanthamoeba*

#### 2.3.1. Traditional classification

Pussard and Pons (1977) classified *Acanthamoeba* cysts into three categories (I-III) according to their size and morphological characteristics. This was the most appropriate classification method at that time, but has been replaced by modern advances in DNA sequencing. However, at least 30 species of *Acanthamoeba* with clear names have been classified in this manner (Table 1).

**TABLE 1:** The traditional classification of *Acanthamoeba*.

Classification	Species	Genotypes	ATCC #	References
Group I	*A. astronyxis*	T7	30137	Ray and Hayes, 1954
	*A. tubiashi*	T8	30867	Lewis and Sawyer, 1979
	*A. comandoni*	T9	30135	Pussard, 1964b
	*A. byersii*	T18	PRA-411	Qvarnstrom et al., 2013
	*A. echinulate*	T4	50239	Pussard and Pons, 1977
Group II	*A. castellanii*	T4	50374 = 30011	Douglas, 1930
	*A. polyphaga*	T4	30871	Page, 1967
	*A. triangularis*	T4	50254	Pussard and Pons, 1977
	*A. rhysodes*	T4	30973	Singh and Das, 1970
	*A. lugdunensis*	T4	50240	Pussard and Pons, 1977
	*A. quina*	T4	50241	Pussard and Pons, 1977
	*A. mauritaniensis*	T4	50253	Pussard and Pons, 1977
	*A. diuionensis*	T4	50238	Pussard and Pons, 1977
	*A. paradiuionensis*	T4	50251	Pussard and Pons, 1977
	*A. griffinii*	T3	30731	Sawyer, 1971
	*A. pearcei*	T3	50435	Nerad et al., 1995
	*A. stevensoni*	T11	50388	Sawyer et al., 1993
	*A. hatchetti*	T11	30730	Sawyer et al., 1977
	*A.* micheli	T9	–	Corsaro et al., 2015
	*A. pyriformis*	T21	–	Tice et al., 2016
	*A. terricola*	T4	30134	Pussard, 1964a
	*A. gigantean*	–	50670	Schmoller, 1964
Group III	*A. healyi*	T12	30866	Moura et al., 1992
	*A. culbertsoni*	T10	30171	Singh and Das, 1970
	*A. lenticulata*	T5	30841	Rivera et al., 1987
	*A. pustulosa*	T2	50252	Pussard and Pons, 1977
	*A. palestinensis*	T2	30870	Reich, 1935
	*A. royreba*	T4	30884	Willaert et al., 1978
	*A. sohi*	–	–	Im and Shin, 2003
	*A. jacobsi*	T15	30732	Sawyer and Nerad, 1992; Hewett et al., 2003

Group I: This group includes five clearly named *Acanthamoeba* listed in Table 1. The average diameter of cysts of this group is greater than or equal to 18 μm. The morphological defining features are that the internal cyst wall is separated from the external wall, the outer cyst wall is slightly wrinkled or smooth, and the internal cyst is often star-shaped (Kong, 2009).

Group II: This group has relatively smaller cysts with an average diameter of less than 18 μm. The outer cyst wall is folded or wavy, and the inner cyst wall is various in shape, which is wavy, round or oval, as well as star-shaped, triangular or tetragonal. The inner wall and outer wall of the cyst are obviously separated and some are closely connected. This group is the most widely isolated *Acanthamoeba*, and most pathogenic *Acanthamoeba* belong to this group (Kong, 2009; Corsaro, 2020). So far, there are 17 species of *Acanthamoeba* which have been clearly named in this group listed in Table 1.

Group III: At present, eight species have been clearly named in this group. This group *Acanthamoeba* cysts are also small and the average diameter is less than 18 μm. The inner wall is round or slightly angular while the outer wall is thin and close to the inner wall, so the outer wall is sometimes difficult to observe (Kong, 2009; de Lacerda and Lira, 2021).

The morphology of *Acanthamoeba* cysts depends upon growth medium used to culture them (Stratford and Griffiths, 1978), while the *Acanthamoeba* cysts in the same group are similar, sometimes there is only a temporary difference between the two kinds of *Acanthamoeba* cysts, therefore, the morphological distinction is subjective and a more scientific classification method with clinical application value was needed.

#### 2.3.2. 18S ribosomal RNA (18S rRNA) gene sequence typing

Gast et al. (1996) began classifying *Acanthamoeba* according to 18S rRNA sequencing. The analysis of full-length 18S rRNA of *Acanthamoeba* is a fast and reliable identification method which is now used extensively to identify *Acanthamoeba* isolates (Maciver et al., 2013). However, the 18S-based genotyping was rendered easier and more rapid by targeting smaller regions of the gene, such as the 464 bp long *Acanthamoeba*-specific amplimer (ASA.S1) which contains the diagnostic fragment 3 (DF3) (Taher et al., 2018). The DF3 region is a 280 bp long single highly variable region within the ASA.S1 region, which is widely used for genotyping studies since it provides equivalent results as that of ASA.S1 (Booton et al., 2002). Each genotype exhibits at least 5% sequence divergence as the typing standard (Khan, 2006). Using this technology, Maghsood et al. (2005) proposed to subdivide T2 into further two groups, i.e., T2a and T2b. T4 is further subdivided into seven groups, T4A, T4B, T4C, T4D, T4E, T4F, and T4G (Corsaro, 2020; Putaporntip et al., 2021). Indeed, DNA sequencing helps to differentiate pathogenic and non-pathogenic isolates within a genotype (Khan, 2006). Currently, most of the diagnostic and epidemiological investigations are indeed carried out with this fragment. Albeit rarely misidentifications may occur, and moreover, reliability of short fragments remains to be discussed especially for phylogenetic analyses and identify new genotypes. Therefore, the full-length gene sequences are strongly recommended (Corsaro and Venditti, 2011; Corsaro et al., 2015).

**FIGURE 3:**
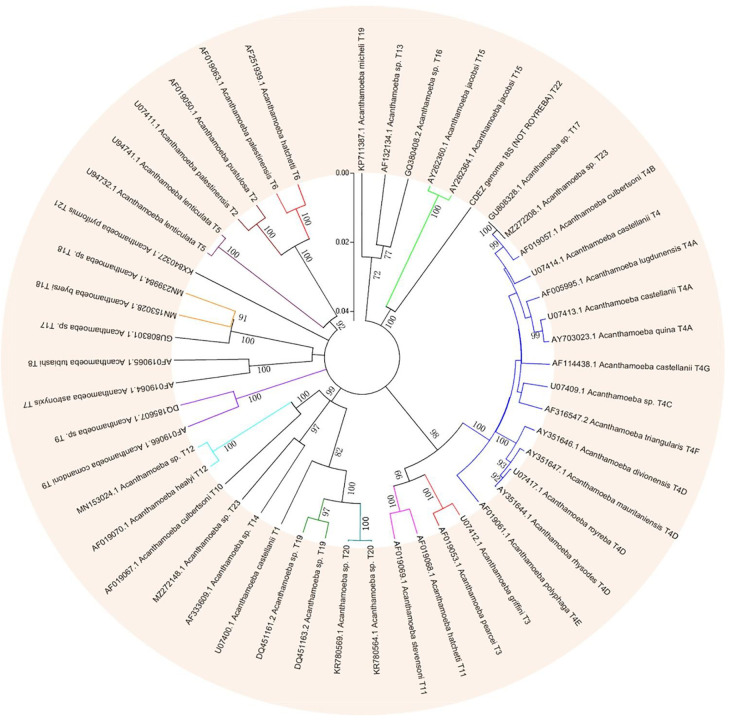
Phylogenetic relationship of 49 various genotypes or subtypes of *Acanthamoeba* T1-T23 based on “complete” 18S rRNA gene sequence. The tree was constructed using the Neighbor-Joining algorithm in MEGA 4.

Based on this method, all *Acanthamoeba* isolates found to date have been divided into 23 genotypes (T1-T23) (Corsaro et al., 2015, 2017; Taher et al., 2018; Corsaro, 2021; Putaporntip et al., 2021). Studies have shown that *Acanthamoeba* genotypes are related to pathogenicity (Walochnik et al., 2000), and the diseases and pathological characteristics caused by different genotypes are unique (Corsaro, 2021). *Acanthamoeba* isolated from patients with the most severe clinical *Acanthamoeba* infections belong to the T4 genotype (Booton et al., 2005; Maciver et al., 2013; Walochnik et al., 2015) followed by the T3 genotype (Booton et al., 2002; Walochnik et al., 2015). There are also reports of *Acanthamoeba* keratitis (AK) caused by T2, T5, T6, T10, T11, T12 and T15 genotypes (Maghsood et al., 2005; Di Cave et al., 2009; Nuprasert et al., 2010; Lorenzo-Morales et al., 2011; Roshni Prithiviraj et al., 2020; Otero-Ruiz et al., 2022) and GAE caused by T1, T2, T5, T10, T12, and T18 (Khan, 2006; Lackner et al., 2010; Qvarnstrom et al., 2013; Duggal et al., 2017; Matsui et al., 2018; Otero-Ruiz et al., 2022). It is difficult to classify the rarer species of *Acanthamoeba*. At present, gene sequencing and morphological classification are often used simultaneously (Walochnik et al., 2000; Kong, 2009). In terms of distribution, the T4 genotype was the most prevalent worldwide, followed by T3, T15, T11, and T5. Furthermore, the T4 genotype contains a higher number of species (Diehl et al., 2021). The currently known *Acanthamoeba* genotypes and corresponding diseases are summarized in Table 2. Meanwhile, phylogenetic relationship with 49 various genotypes or subtypes of *Acanthamoeba* T1-T23 based on “complete” 18S rRNA gene sequence has been shown in Figure 3.

**TABLE 2:** Known *Acanthamoeba* genotypes and associations with human diseases.

*Acanthamoeba* genotypes	Human diseases		
T1	–	GAE	
T2	AK	GAE	–
T3	AK	–	–
T4	AK	GAE	–
T5	AK	GAE	–
T6	AK	–	–
T7	AK (rare)	–	–
T8	AK	–	–
T9	AK (rare)	–	–
T10	AK	GAE	–
T11	AK	–	–
T12	–	GAE	–
T13	AK	–	–
T14	–	–	–
T15	AK	–	–
T16	AK (rare)	–	AP
T17	–	–	–
T18	–	GAE	–
T19	–	–	–
T20	–	–	–
T21	–	–	–
T22	–	–	–
T23	–	–	–

## 3. *Acanthamoeba* related diseases

### 3.1. Disease type

#### 3.1.1. *Acanthamoeba* keratitis

*Acanthamoeba* keratitis (AK) is the most common disease caused by *Acanthamoeba* (Stehr-Green et al., 1989; Ibrahim et al., 2007; Jiang et al., 2015). The incidence of AK correlates significantly with corneal trauma, exposure to sewage, and wearing of contact lenses (CLs) (Moore et al., 1985; Maycock and Jayaswal, 2016; Hassan et al., 2019). Wearing CLs can cause slight abrasion on the cornea, which destroys the integrity of corneal epithelium and increases the chance of invasion of pathogenic microorganisms (Ibrahim et al., 2009). *Acanthamoeba* adhesion to the surface of contact lenses is one of the essential first steps in the pathogenesis of AK (Lee et al., 2016) and it was found that the adhesion rate of trophozoites to CL material was higher than that of cysts (Kilvington, 1993). Even when exposed to a minimally contaminated solution or environment for a few seconds, trophozoites immediately show adherence to CLs (John et al., 1989; Heidemann et al., 1990). Later, silicone hydrogel lenses became popular, accounting for 30% of new lenses in 2005 (Morgan et al., 2006). However, the first generation of silicon hydrogel lenses were found to be highly sticky to trophozoites (Beattie et al., 2003). To overcome this issue, second generation silicon hydrogel lenses have been developed and trophozoite attachment rates for this generation of lenses were much lower than for the first generation and not statistically different from those of conventional hydrogel lenses (Beattie et al., 2006). Recently, Campolo et al. (2022) found that some contact lens materials are more conducive to cyst formation than the natural environment with encystation occurring within as little as 4 h of incubation. They hypothesized that aggregation of cysts directly obstructs multi-purpose solutions from disinfecting *Acanthamoeba*, which further indicates that contact lens materials may need to be reevaluated in the future as infection incidence increases (Campolo et al., 2022). The adhesion of trophozoites and cysts to the surface of CLs depends on the structure of both. Pseudopods of trophozoites play an important role in adhesion (John et al., 1989; Omaña-Molina et al., 2014), but cysts do not have pseudopod structure. Instead, adhesion occurs through adhesive substances in the ectocyst (Kilvington, 1993). Infection by *Acanthamoeba* starts on the epithelium and progresses slowly into the stroma (Marciano-Cabral and Cabral, 2003). In most cases, AK is unilateral, however, evidence also suggests that it can affect both eyes (Voyatzis and McElvanney, 2007; Lee and Gotay, 2010). Symptoms of AK include conjunctival hyperemia, edema, tearing, foreign body sensation, blurred vision, decreased visual acuity, photophobia, and severe ocular pain (Kaiserman et al., 2012; Lorenzo-Morales et al., 2013, 2015). When treatment is inadequate, AK can cause corneal perforation and melting (Lorenzo-Morales et al., 2015). The process of AK infection is complex and requires both host and pathogen factors discussed later in the text.

Poor CL hygiene habits are also risk factors to induce AK (Ahearn and Gabriel, 1997; Cope et al., 2015). Studies have shown that *Acanthamoeba* exist in tap water (Carnt et al., 2020); therefore, rinsing and cleaning lenses with tap water before putting them in the storage case may cause contamination of lenses and cause infection (Nau and Dhaliwal, 2018). Repeated use of lens disinfectant by “topping off” old solution with new disinfectant should be avoided (Tu and Joslin, 2010). Additionally, the use of expired solution (Abjani et al., 2016), the use of self-made saline solution, and chlorine-based disinfection can increase the risk of disease (Schaumberg et al., 1998; Seal et al., 1999; Radford et al., 2002). In general, the long-term use of the same CL storage case and insufficient drying after cleaning are a breeding ground for bacteria, protozoa, and fungi which themselves are a food source of trophozoites (Kuzman et al., 2014; Zimmerman et al., 2017; Mirsayafov et al., 2018; Tan et al., 2018). Bad habits also include rubbing the eyes while wearing CLs, leading to corneal damage and promoting the invasion of *Acanthamoeba* (Taher et al., 2018). Other risk factors include sleeping with contact lenses that can lead to corneal hypoxia, edema, thinning in the center of the cornea (Ibrahim et al., 2009), and superficial punctate keratitis (Taher et al., 2018).

The symbiotic relationship of bacteria and *Acanthamoeba* (Rayamajhee et al., 2022) may increase corneal damage after *Acanthamoeba* infection. The ability of bacteria to survive as an amoebal endosymbiont was first reported in 1975 (Proca-Ciobanu et al., 1975), while the role of *Acanthamoeba* as a host of pathogenic microorganisms was reported in 1978 (Prasad and Gupta, 1978). Spores of *Bacillus anthracis* (Ames strain) can germinate in the amoeba phage, and the number of spores can increase 50-fold after 72 h (Dey et al., 2012). *A. castellanii* can also host *Legionella* (Swart et al., 2018; Nomura et al., 2022), *Vibrio cholerae*, *Francisella tularensis* (Abd et al., 2003) and the causative agent of Johne’s disease, *Mycobacterium avium* subsp. *paratuberculosis* (MAP) (Phillips et al., 2020). These bacteria have been referred to as amoeba-resistant bacteria. *A. castellanii* releases undigested *V. cholerae* in expelled food vacuoles (EFVs) in the form of free spherical pellets (1–5 μm size) (Espinoza-Vergara et al., 2020). If given the right amount of nutrients or cultured at 37°C, hundreds of bacteria can escape from EFV (Rayamajhee et al., 2022). Therefore, the difficulty of treatment can increase dramatically in opportune situations.

#### 3.1.2. Granulomatous amoebic encephalitis

Granulomatous amoebic encephalitis (GAE) caused by *Acanthamoeba* infection is a rare central nervous system disease that is highly fatal with mortality rate greater than 90% despite its low occurrence worldwide (Kot et al., 2018; Kalra et al., 2020). GAE is a subacute or chronic granulomatous encephalitis characterized by neck ankylosis, headache, and fever. Other progressive neurological symptoms include altered mental state, seizures, confusion, hallucination, focal neurologic signs, diplopia, cranial nerve palsies, ataxia, high grade flaccid paralysis of right lower limb, lethargy, stiff neck, and personality changes (Coven et al., 2017; Ghadage et al., 2017; Geith et al., 2018). GAE is a progressive disease leading to death within 1–2 months of symptom onset due to increased intracranial pressure (Duggal et al., 2017). It occurs especially in immunocompromised individuals including those infected with human immunodeficiency virus (HIV) or acquired immunodeficiency syndrome (AIDS), organ transplant recipients, patients with diabetes, systemic lupus erythematosus (SLE), those undergoing cancer treatment as well as immunocompetent individuals (Lalitha et al., 1985; Gonzalez et al., 1986; Carter et al., 2004; Vernon et al., 2005; Duarte et al., 2006; Sütçü et al., 2018). The pathogenesis of GAE is not fully understood. *Acanthamoeba* may enter through various routes including lower respiratory tract or breaks in the skin resulting in hematogenous dissemination to the brain (Duggal et al., 2017). Although there is no clinical evidence, *Acanthamoeba* is likely to enter the central nervous system through the blood-brain barrier and cause infection. It is worth noting that the olfactory epithelium may be another pathway for *Acanthamoeba* to enter the central nervous system (Kalra et al., 2020). GAE cases are often under diagnosed and hence strong clinical suspicion along with laboratory technical expertise is required for early diagnosis and therapeutic intervention (Parija et al., 2015).

#### 3.1.3. Other diseases

*Acanthamoeba* can also cause a skin disorder known as cutaneous acanthamoebiasis (CA) and pulmonary infection, *Acanthamoeba* pneumonia (AP), but both conditions are rare. The first case of cutaneous *Acanthamoeba* infection in an AIDS patient was reported in 1986 (Gonzalez et al., 1986). Eighteen patients with AIDS and cutaneous acanthamoebiasis have been reported in the literature to date (Torno et al., 2000). A recent article of *Acanthamoeba* infections identified a number of symptoms, including fever, headache, dizziness, nausea, altered mental status, seizures, and facial palsies prior to cutaneous manifestations (Zhang and Cheng, 2021). Lesions frequently occur on the face and extremities and exhibit heterogenous morphology, ranging from papules, pustules, nodules, ulcers, eschars, or abscesses (Murakawa et al., 1995). However, diagnosing cutaneous acanthamoebiasis is challenging given its variable clinical presentation and lack of pathognomonic findings (Kutner et al., 2018).

*Acanthamoeba* pneumonia occurs mostly in patients with a low immune response (Visvesvara et al., 1983; Kaul et al., 2008). So far, 19 case reports of *Acanthamoeba* pneumonia (AP) or disseminated acanthamoebiasis with lung infection have been published. Most patients came from the USA, but there were also cases from Poland, Austria, France, Korea, Japan, and India. None of the patients survived (Kot et al., 2021). In patients with AP, a decrease in body weight and respiratory efficiency was observed, and in the radiological examination, interstitial changes with visible pulmonary edema were observed (Im and Kim, 1998; Kot et al., 2021).

### 3.2. Pathogenic mechanism

Among the diseases caused by *Acanthamoeba*, the pathogenic mechanism of the rare GAE, CA, and pulmonary infection is not apparent and critically understudied compared to the relatively common AK infection. Once *Acanthamoeba* adheres to the target cells, intracellular signal transduction is quickly activated and a series of cascade effects are triggered including phagocytosis of target cells, secretion of protease, and induction of apoptosis, which will cause direct pathological damage to the host. These are further explored in-depth below.

#### 3.2.1. Adhesion

The ability of *Acanthamoeba* to bind to epithelial cells is the basis of infection. At present, it is believed that there are two main factors involved in this process, one is acanthopod, the other is surface adhesion. These two factors are very important with adhesion ability of *Acanthamoeba* and directly correlate to the pathogenicity of different isolates. Studies in recent years have found that the total number of acanthopods is closely related to the adhesion rate. Pathogenic *Acanthamoeba* has more than one hundred acanthopods per cell, whereas non-pathogenic *Acanthamoeba* has no more than twenty acanthopods per cell (Lorenzo-Morales et al., 2013).

Current consensus is that the adhesion of pathogenic *Acanthamoeba* to corneal epithelial cells is mainly mediated by adhesins, including mannose-binding protein (MBP) and laminin- binding protein (LBP) (Huth et al., 2017; Corsaro, 2022). MBP is expressed by *Acanthamoeba* protozoa which is composed of several 130 kDa subunits distributed on the surface of *Acanthamoeba* and can be isolated and extracted by mannose affinity column affinity chromatography (Garate et al., 2006a,b). *Acanthamoeba* MBP is a transmembrane protein encoded by a gene containing six exons and five introns and has a typical cell surface receptor function (Garate et al., 2004). As an exogenous lectin, it is generally bound to specific mannose-containing glycoproteins through the carbohydrate recognition domain (CRD) (Garate et al., 2006a). MBP binds to two glycoproteins of corneal epithelial cells, which are purified mannose protein and mannose bovine serum albumin (isolated and identified from primary cell cultures of rabbit corneal epithelium) (Yang et al., 1997). This is further demonstrated by studies showing that binding can be inhibited by exogenous α-mannose competitively (Rocha-Azevedo et al., 2010; Kim et al., 2012), while galactose bovine albumin and other non-competitive sugars could not inhibit MBP binding, indicating that mannose’s inhibitory effect was achieved by competing with MBP for sugar binding sites (Rocha-Azevedo et al., 2010). The binding of *Acanthamoeba* and mannose-containing glycoproteins leads to an increase of serine protease secretion, which is the decisive factor in host cell injury (Rocha-Azevedo et al., 2010; Lorenzo-Morales et al., 2013). MBP-mediated adhesion of *Acanthamoeba* to host cells depends on the level of MBP expression on the surface of *Acanthamoeba* and the number of mannose-containing glycoproteins synthesized by host cells. The study found that the damaged cornea can expose more mannose-rich glycoproteins, the number of *Acanthamoeba* attached to its surface is higher than healthy corneas. In addition, compared with non-pathogenic *Acanthamoeba*, pathogenic strains showed higher MBP expression levels (Yoo and Jung, 2012; Huth et al., 2017). Currently two types of MBP (MBP1 and MBP2) have been found (Corsaro, 2022). MBP1 is a conventional membrane protein with a signal peptide at the N-terminus and a transmembrane domain located at the C-terminus. The extracellular portion contains a Cys-rich repetitive motif (CXCXC) and a domain of unknown function (DUF 4114), while two NPLF motifs involved in intracellular signaling are located in the intracytoplasmic region (Garate et al., 2004). MBP1 appears to be specific only to *Acanthamoeba* species of groups 2 and 3, with different gene structure and amino acid sequence depending on the genotype, while shorter MBP-like sequences could be identified in the group 1 species (*A. astronyxis* T7 and *A. byersi* T18), as well as in T4 and T2 genotypes. The resulting protein, labeled MBP2, covers the N-terminal part containing DUF 4114 but lacks the Cys-rich repetitive elements (usually only a single CXCXC motif is present), as well as the intracytoplasmic domain (Corsaro, 2022). MBP2 has a signal peptide at the N-terminal followed by a transmembrane motif, although a second short transmembrane motif is predicted at the C-terminus for group 1 species (Corsaro, 2022). *In silico* alignment of two kinds of MBP (L8GXW7, 360 aa; Q6J288, 833 aa) demonstrated 19.4% identity (94 similar positions). Both proteins also share a domain of unknown function (DUF4114), sharing 61.6% identity (L8GXW7, 164–256 aa and Q6J288, 156–254 aa; 61 identical positions) (Gonçalves et al., 2019). MBP1 sequences from different genotypes are variable, with identity/similarity values < 60/75%. Moreover, values between MBP1 and MBP2 are even lower (between 25 and 35%), the most conserved region being the DUF4114 domain (approximately 65% of identical sites) (Corsaro, 2022).

In addition to MBP, another important *Acanthamoeba* adhesin is the laminin-binding protein (LBP), which allows further progression of infected tissues (Rocha-Azevedo et al., 2009), as laminin is a major glycoprotein of the extracellular matrix separating epithelia from other tissues. The molecular weight is predicted to be 28.2 kDa (Hong et al., 2004; Omaña-Molina et al., 2017) and 55 kDa in *A. culbertsoni* (Rocha-Azevedo et al., 2009, 2010). *Acanthamoeba* LBP belongs to the family of non-integrin 37/67-kDa laminin receptors (37/67LR), also involved as receptors for viruses and other pathogens as well as in other cellular processes such as motility and differentiation (DiGiacomo and Meruelo, 2016). LBP homologs are present in all organisms including prokaryotes as this adhesin derives from a 40S ribosomal protein which evolved the ability to bind laminin (Ardini et al., 1998). Overall, LBPs have a short transmembrane domain at the N-terminal, three recognition domains for laminin on the extracellular C-terminal domain, comprising a palindromic LMWWML motif located in the peptide G (Castronovo et al., 1991), a direct binding region (DBR), and TEDWS motif repeats. LBP sequences are highly conserved with identity/similarity values > 80/90% for those of group 2 and 3 species and around 60/70% between these and those of group 1 species (Corsaro, 2022). It is generally believed that the adhesion of *Acanthamoeba* to the cornea is a crucial prerequisite for the subsequent inflammatory response and the degree of adhesion is directly proportional to the strength of the host’s inflammatory response (Wang and Zhu, 2010). LBP participates in the initial phase where infiltration is limited to the corneal epithelium, particularly in the intercellular space (Gu et al., 2022). Therefore, the selectivity of *Acanthamoeba* for the host cornea also determines the differences in the specificity of AK in different hosts. A similar view has been established in the pathogenic mechanism of other protozoa such as the binding and lysis of *Entamoeba histolytic* to host cells being mediated by galactose adhesion protein (Guzmán-Téllez et al., 2020). Studies shows that expression levels of both MBP and LBP vary between *Acanthamoeba* strains and correlate with pathogenicity (Garate et al., 2006b; Ng et al., 2017). They were found in either low or non-existent quantities in non-pathogenic *Acanthamoeba* (Rocha-Azevedo et al., 2009; Singh et al., 2012; Huth et al., 2017; Corsaro, 2022).

#### 3.2.2. Phagocytosis

Phagocytosis plays an essential role in the pathogenesis of *Acanthamoeba* infection. Phagocytosis is an actin-dependent process that drives cytoskeleton rearrangement. Cytochalasin D (a toxin that blocks actin polymerization) inhibits *Acanthamoeba*-mediated host cell death, confirming that actin-mediated cytoskeleton rearrangement plays an important role in *Acanthamoeba* phagocytosis (Taylor et al., 1995; Niederkorn et al., 1999; Alsam et al., 2005a). In order to study the relationship between cellular signal transduction pathway and phagocytosis, phagocytosis assays have been performed in the presence of protein tyrosine kinase inhibitor, genistein and a protein tyrosine phosphatase inhibitor, sodium orthovanadate. *Acanthamoeba* uptake of *Escherichia coli* is significantly reduced in the presence of genistein. In contrast, sodium orthovanadate increases bacterial uptake by *Acanthamoeba* (Alsam et al., 2005a). Rho GTPases are the key regulators of the actin cytoskeleton in all eukaryotic cells and link external signals to cytoskeleton (Mackay and Hall, 1998). This process involves three major pathways including RhoA, Rac1, and Cdc42 (Mackay and Hall, 1998). The Rho kinase inhibitor, Y27632, which partially blocks RhoA pathway, reduced bacterial uptake (Alsam et al., 2005a). These results suggested that the signal transduction pathway may regulate phagocytosis through mediating actin polymerization. Some evidence suggests that phosphatidylinositol 3-kinase (PI3K) may play important roles in regulating actin dependent-processes (Wymann and Pirola, 1998). For example, studies have shown that PI3K controls Rho-mediated changes in actin cytoskeleton in fibroblasts (Reif et al., 1996; Cantrell, 2001). When studying the phagocytosis of *Acanthamoeba*, it was found that LY294002, a specific inhibitor of PI3K, could significantly reduce the phagocytosis of *E. coli* (Alsam et al., 2005a). Interestingly, studies have also shown that the involvement of PI3K in Rac1-dependent lamellipodia formation (Wennström et al., 1994) and Cdc42-dependent cytoskeletal changes (Jiménez et al., 2000), suggesting that other GTPases such as Cdc42 and Rac1 are also involved in the phagocytosis of *Acanthamoeba*.

In addition, there is a unique actin-rich sucking structure (amoebostomes) on the surface of *Acanthamoeba*, also known as the food cup structure, which is a temporary structure that mediates phagocytosis of bacteria, yeasts, or cells (Pettit et al., 1996; Marciano-Cabral and Cabral, 2003). The mechanism involved in these goblet structures is called “trogocytosis,” which is the process whereby sections of host cells are ripped off by the amoeba (Nakada-Tsukui and Nozaki, 2021). Trogocytosis can be regarded as a special type of phagocytosis. The initial discovery of cell nibbling is from unicellular eukaryotes (Nakada-Tsukui and Nozaki, 2021). Culbertson (1970, 1971) described trogocytosis-like events in two species, *Naegleria fowleri* HB-1 and *Hartmannella*-*Acanthamoeba* A-1. When these amoebae were inoculated into guinea pigs, pathological examination showed that amoeba in thrombi internalized erythrocytes only halfway (i.e., trogocytosis) (Culbertson, 1971). Later, trogocytosis of a mouse embryonic cell by *N. fowleri* was also confirmed (Brown, 1979). Petti found that trophozoites of 4 species of *Acanthamoeba* were cytopathic for cultured rat B 103 neuroblastoma cells and this process is achieved by destroying nerve cells through the food cup (Pettit et al., 1996). The same process can be observed in the pathogenesis of other protozoa such as *N. fowleri* (Tiewcharoen et al., 2008) and *E. histolytica* (Miller et al., 2019). In the study of *E. histolytica*, it was found that there was pronounced actin polymerization within the amoebae at the site of human cell contact and ingested fragments were surrounded by polymerized actin, which indicates that trogocytosis is related to actin rearrangement (Ralston et al., 2014). Further studies found that Gal/GalNAc lectin, EhC2PK, and PI3K signaling were also involved in the amoebic trogocytosis-mediated human cell killing (Ralston et al., 2014; Miller et al., 2019).

In recent years, some additional proteins that play a role in *Acanthamoeba* phagocytosis have been reported. For example, *Acanthamoeba* SBDS (Shwachman-Bodian-Diamond Syndrome)-related proteins are highly expressed during phagocytosis, which may be related to cytoskeleton-related phagocytosis and cystogenesis (Wang et al., 2021). Studies have shown that *Acanthamoeba* secreted extracellular M20/M25/M40 superfamily aminopeptidase plays an important role in the *Acanthamoeba* pathogenesis (Huang et al., 2017). *Acanthamoeba* Type-I metacaspase (Acmcp) is a caspase-like protein which is expressed during the encystations of *A. castellanii*. When vectors containing Acmcp (pDneo2a-GFP-Acmcp) were electro-transfected into wild type *Dictyostelium discoideum* cells, they showed a significant increase in the fluid phase internalization and phagocytosis rate compared to the control cells (Saheb et al., 2015). Therefore, metacaspase is proposed as a candidate drug target against infections caused by *A. castellanii*. Finally, *A. castellanii* Rab7 (AcRab7), which is involved in endosomal delivery after phagocytosis and dominates energy production and cell growth, may be an important target in some species (Hong et al., 2022).

**FIGURE 4:**
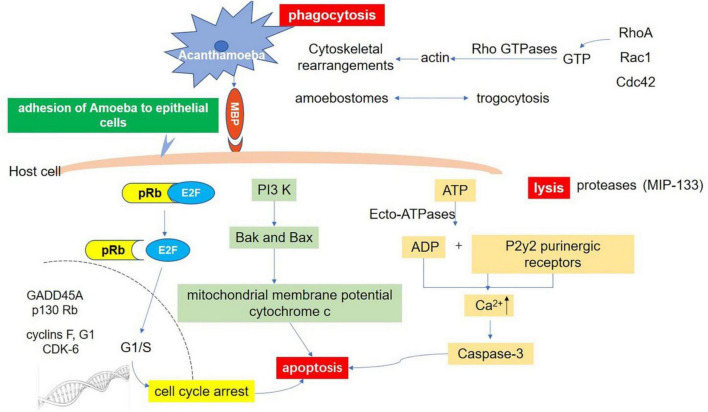
Host-Parasite interaction molecular signaling pathways. Adhesion through spinous pseudopodia and adhesin (such as MBP) is the basis for *Acanthamoeba* to establish infection. Once adhesion is completed, the intracellular signal transduction process is activated and further triggers cascade effects such as phagocytosis of target cells, secretion of protease, and apoptosis, resulting in direct pathological damage. Phagocytosis: There are three fully studied pathways involved in this process. The RhoA pathway, which leads to stress fiber formation; Rac1 activation, which triggers plate foot formation; and Cdc42 activation, which promotes filamentous foot formation. Apoptosis: There are at least three pathways related to apoptosis after infection of *Acanthamoeba*. Ecto-ATPases driven cell apoptosis; Interference with the expression of important genes that regulate cell cycle; and the phosphatidylinositol 3-kinase (PI3K) mediated apoptosis pathway. Lysis: Acanthamoeba secretes a variety of proteases involved in cell lysis. A serine protease (MIP133) has been identified as a key component in the pathogenesis of *Acanthamoeba*.

It has also been reported that mannose inhibits *Acanthamoeba* phagocytosis, which suggests that *Acanthamoeba* phagocytosis is a receptor-dependent process and *Acanthamoeba* adhesin (or MBP) is involved in phagocytosis (Allen and Dawidowicz, 1990). Further study of other molecular pathways and the interaction between various intracellular signaling pathways will help the field to understand the phagocytosis of *Acanthamoeba* and provide a basis for clinical treatment. *Acanthamoeba*-mediated host signaling pathways as well as self-signaling pathways are shown in Figure 4.

#### 3.2.3. Apoptosis of host cells

In addition to directly causing cell death, *Acanthamoeba* can induce programmed cell death, such as apoptosis. After *Acanthamoeba* infection, cell membrane rupture, condensation of nucleoplasm, and degradation of DNA can be seen, eventually forming apoptotic bodies (Khan, 2006; Lorenzo-Morales et al., 2013). Based on current research, there are at least three pathways related to apoptosis. First, Ecto-ATPase driven cell apoptosis. Ecto-ATPases hydrolyze extracellular ATP and other nucleoside triphosphates and the resulting ADP has a toxic effect on host cells. It has been shown that the ADP released binds to the P2y2 purinergic receptor on the host cell, resulting in the increase of intracellular calcium and the activation of caspase-3, which eventually leads to cell apoptosis (Mattana et al., 2002). Suramin, a P2 receptor antagonist, inhibits *Acanthamoeba*-mediated host cell apoptosis, indicating that extracellular ATP enzymes play an essential role in the pathogenesis of *Acanthamoeba* (Mattana et al., 2002). Compared with weakly pathogenic isolates, clinical isolates of *Acanthamoeba* show higher extracellular ATP activity (Sissons et al., 2004a). Several Ecto-ATPases with estimated molecular weights of 62, 100, 218, 272, and exceeding 300 kDa are described in *Acanthamoeba* (Sissons et al., 2004a). However, further studies are needed to clarify their function in *Acanthamoeba* biology and their pathogenicity to the host. Secondly, *Acanthamoeba* causes cell cycle arrest by affecting the expression of GADD45A, p130Rb, F, G1 protein, and cyclin-dependent kinase 6. The dephosphorylation of retinoblastoma protein (pRb) further supports this theory (Sissons et al., 2004b). Unphosphorylated pRb binds to the E2F transcription factor during the cell cycle, which inhibits E2F entry into the nucleus. When phosphorylated by CDKs, the conformational change of pRb leads to the dissociation of the pRb-E2F complex. The released E2F enters the nucleus and initiates the synthesis of DNA (Dyson, 1998; Harbour and Dean, 2000; Stevaux and Dyson, 2002). Recent studies have found that *Acanthamoeba* can induce cell cycle arrest of host cells by inhibiting this process in human corneal epithelial cells and brain microvascular endothelial cells (Sissons et al., 2004b). Third, *Acanthamoeba*-mediated apoptosis of host cells depends on the activation of phosphatidylinositol 3-kinase (PI3K) (Sissons et al., 2005). This conclusion was confirmed by using LY294002 to inhibit the activity of PI3K specifically and to express the mutant form of PI3K regulatory subunit P110 in host cells (Sissons et al., 2005). Traditionally, it has been known that PI3K plays an important role in regulating cell survival pathways. For example, Thyrell et al. (2004) have shown that α-interferon (IFNa) can induce PI3K-mediated apoptosis in myeloma cells without Akt phosphorylation. Further studies have shown that PI3K can mediate apoptosis by activating the downstream pre-apoptotic molecules Bak and Bax, inducing the loss of mitochondrial membrane potential and the release of cytochrome c (Mattana et al., 2002; Chusattayanond et al., 2010). A similar mechanism may exist in the process of host cell apoptosis mediated by *Acanthamoeba*.

#### 3.2.4. Lysis

When *Acanthamoeba* adheres to the host cells, it secretes a variety of proteases that create pores in host membranes, resulting in the lysis of cells and tissues (Lorenzo-Morales et al., 2013). Pathogenic *Acanthamoeba* shows higher extracellular protease activity. Protease-mediated lysing plays an important role in the pathogenesis of various protozoa, such as amoeba, trichomonas, leishmania, trypanosoma, and malaria parasites, and is directly involved in cell and tissue invasion and damage (Serrano-Luna Jde et al., 2006). It was found that *Acanthamoeba* mainly produce three types of proteases: serine proteases, cysteine proteases, and metalloproteinases (Khan, 2006). Serine proteases are the most abundant and present in almost all *Acanthamoeba* genotypes (Serrano-Luna Jde et al., 2006; Cirelli et al., 2020). Several serine proteases with molecular weights ranging from 20 to 200 kDa have been identified. They not only have collagen degradation activity, but also can degrade plasminogen activator, fibrinogen, IgG, IgA, albumin, hemoglobin, protease inhibitor, and interleukin-1 (Na et al., 2001, 2002). A serine protease called MIP133 has been identified as a key component in the pathogenesis of *Acanthamoeba*. MIP133 serine protease can induce the lysing of corneal cells, iris ciliary body cells, retinal pigment epithelial cells, corneal epithelial cells, and corneal endothelial cells as well as induce apoptosis of macrophage-like cells (Lorenzo-Morales et al., 2015). The direct damage of serine protease in *Acanthamoeba* infection can be observed by injecting *Acanthamoeba* into the corneal stroma. This damage can be inhibited by the serine protease inhibitor phenylmethanesulfonyl fluoride (Na et al., 2001, 2002). In addition, siRNA targeting the catalytic domain of serine proteases reduced protease activity and host cytotoxicity mediated by *Acanthamoeba* (Lorenzo-Morales et al., 2005). The protease activity of pathogenic isolates, especially serine proteases, is significantly higher than that of non-pathogenic isolates, which is consistent with their cytotoxic effect on host cells.

Cysteine proteases with different molecular weights are detected in *Acanthamoeba* cell lysate and culture supernatant. These enzymes are thought to be involved in cell degradation (Hadas and Mazur, 1993; Mitro et al., 1994; Alfieri et al., 2000; Leitsch et al., 2010; Moon et al., 2012b; Ramírez-Rico et al., 2015). For example, two L-cysteine proteases, AcCP and AhCP, were identified in *Culbertson* and *Healyi Acanthamoeba* (Yun et al., 1999; Hong et al., 2002, 2018). Recombinant AcCP showed enzyme activity under acidic conditions and is the most suitable for pH4.0. Recombinant enzymes can effectively hydrolyze human proteins, including hemoglobin, albumin, immunoglobulin A and G, and fibronectin under acidic conditions (Ramírez-Rico et al., 2015; Hong et al., 2018; Cirelli et al., 2020). In addition, cysteine proteases with molecular weights of 43, 65, 70, and 130 kDa have also been reported (Khan, 2006). Although the histolytic role of cysteine proteases in the pathogenesis of parasitic pathogens has been identified (Singh et al., 2004; Jimenez-Sandoval et al., 2017), the studies on cysteine proteases in *Acanthamoeba* are limited.

In addition to serine and cysteine proteases, there is evidence of metalloproteinase activity in *Acanthamoeba* (Łanocha-Arendarczyk et al., 2018). Metalloproteinases usually play an important role in cell differentiation and migration, regulation of growth factor activity, angiogenesis, and inflammation (Sternlicht and Werb, 2001; Parks et al., 2004). An extracellular metalloprotease of 150 kDa was identified from the T1 genotype isolate of *Acanthamoeba* (Alsam et al., 2005b; Sissons et al., 2006). This metalloprotease exhibits extracellular matrix lytic properties for collagen I and III (the main components of the extracellular collagen matrix), elastin (the elastic fibers of the extracellular matrix), and plasminogen (involved in the extracellular matrix proteolysis). These, as well as casein, gelatin, and hemoglobin are degraded (Sissons et al., 2006). The specific mechanism needs to be further studied.

Phospholipases are known to cleave phospholipids, suggesting their possible involvement in the host cell plasma membrane disruption leading to host cell penetration and lysis. Matin and Jung (2011) tested the phospholipase activity and cytotoxicity of three different *Acanthamoeba* strains including an encephalitis isolate (T1 genotype), a keratitis isolate (T4 genotype), and an environmental isolate (T7 genotype) *in vitro*. The results show that all strains exhibited phospholipase A(2) [PLA(2)] and phospholipase D (PLD) activities. Moreover, *Acanthamoeba* isolates exhibited higher PLD activities compared with the PLA(2). Interestingly, the encephalitis isolates of *Acanthamoeba* exhibited higher phospholipase activities as compared with the keratitis isolates, but the environmental isolates exhibited the highest phospholipase activities, suggesting possible differences in phospholipases in *Acanthamoeba* belonging to different genotypes (Matin and Jung, 2011). The result supporting this inference is that compound 48/80 partially blocked the encephalitis isolate-mediated cytotoxicity, i.e., 49% cell death in the presence of the inhibitor compared with 73% in the absence of the inhibitor, while it had no effect on the keratitis isolate cytotoxicity and the environmental isolate exhibited minimal cytotoxicity even in the absence of inhibitors (Matin and Jung, 2011).

Although phospholipase inhibitors do not clearly block the cytotoxicity mediated by *Acanthamoeba* alone, this does not rule out that they are involved in *Acanthamoeba* pathogenesis. *Acanthamoeba* pathogenesis is a process involved in many factors, including adhesion, phagocytosis, apoptosis, proteolytic enzyme, extracellular ATPase and so on as showing in Figure 4. The inhibition of a single factor may not be sufficient to kill host cells. In support of this notion, previous studies have shown that inhibition of *Acanthamoeba* binding to host cells is not adequate to block host cell cytotoxicity (Leher et al., 1998). It is also possible that cytotoxicity is a delayed event and that phospholipases are involved in the early events. Studies have shown that phospholipases involved in interference with the intracellular signaling pathways. Phospholipases generate lipids and lipid-derived products that act as mediators and second messengers, which may act as the modulators of signal transduction pathways (Dennis et al., 1991; Serhan et al., 1996). Studies have shown that lysophospholipids, a by-product of PLA2 and phospholipase B (PLB), can induce the activation of protein kinase C, which has diverse function in host cell signal transduction pathways (Oishi et al., 1988). Phospholipase C of *Clostridium perfringens* induces expression of IL-8 synthesis in endothelial cells (Bryant and Stevens, 1996; Bunting et al., 1997). These studies suggest that *Acanthamoeba* phospholipase may play a role in causing host cell damage or affecting other cellular functions such as inducing inflammation. In addition, studies on the pathogenicity of other species mediated by phospholipase have also been reported, such as the potential to prevent Candida infection by targeting phospholipase with synthetic compounds (Hänel et al., 1995). Future studies are needed to identify and characterize *Acanthamoeba* phospholipases, which should help determine their potential role for therapeutic interventions and in differentiation of *Acanthamoeba* isolates belonging to different genotypes.

*Acanthamoeba* species also show neuraminidase activity (Pellegrin et al., 1991). *Acanthamoeba* can release sialic acid after invading human cells, so the neuraminidase may be related to its colonization and play an important role in damaging the corneal epithelium rich in sialic acid (Pellegrin et al., 1991). In immunofluorescence, immunoblotting, and enzyme-linked immunosorbent assays, the neuraminidase antibody of *Trypanosoma cruzi* cross-reacted with *Acanthamoeba*, indicating that Acanthamoeba does have neuraminidase activity (Pellegrin et al., 1992). Although the role of *Acanthamoeba* neuraminidase in the pathogenesis of keratitis is unclear, the fact that cell damage can occur without direct contact (Visvesvara and Balamuth, 1975) suggests that the release of parasite products is involved in the mechanism of tissue injury. Neuraminidase-induced cleavage of sialic acid from glycoproteins and gangliosides of cerebral tissue may lead to the onset of granulomatous amoebic encephalitis. Such an effect of neuraminidase was suggested in primary amoebic meningoencephalitis caused by *N. fowleri*, also a free living amoeba (Eisen and Franson, 1987). It is generally accepted that the ability of microorganisms to produce disseminated infections is related to their capacity to destroy colonized tissues. Neuraminidase may be related to the components and enzymes of several other amoebae, such as phospholipase A, sphingomyelinase, elastase, collagenase, and a cytolytic and granule-associated cytotoxic activity. The synergistic effect of these factors may participate in the destruction, mucosal surface penetration, and dissemination of *Acanthamoeba* (Ferrante and Bates, 1988; He et al., 1990).

#### 3.2.5. Immune escape

The immune escape ability of *Acanthamoeba* is also an important component of its pathogenicity. Compared with uninfected individuals, the levels of IgA and IgG in tears of AK patients are lower (Carnt and Stapleton, 2016; Neelam and Niederkorn, 2017), which may be related to the fact that its secreted serine protease can destroy IgA and IgG antibodies in human tears (Kong et al., 2000; Marciano-Cabral and Cabral, 2003; Foulks, 2007). This reduction of antibodies allows *Acanthamoeba* to evade the human immune response and survive long-term in the host. Continuous low-level turnover of complement components within the eye has been recognized for many years (Sohn et al., 2000) and is known to be a major contributor to the immune privilege status of the eye (Niederkorn, 2007). For example, the major component of immune privilege, termed anterior chamber-associated immune deviation (ACAID), has been shown to be complement-dependent (Sohn et al., 2003). The complement cascade has a well-established role in the maintenance of a healthy cornea (Mondino et al., 1996). Although membrane-bound complement regulators such as CD46, CD55, and CD59 are expressed throughout the various layers of the cornea, there is a disproportionately high level of expression in the corneal epithelium (Bora et al., 1993). This may be due to the fact that the corneal surface is often exposed to various pathogens, resulting in the continuous activation of the complement system. Some bacteria produce phospholipases and other enzymes that can remove CD55 and CD59 from the surface of corneal epithelium (Cocuzzi et al., 2000), causing complement regulation disorders, which aggravate bacterial keratitis and even lead to loss of vision (Tang et al., 2009). By comparing the results of systematic analysis of *Acanthamoeba* isolates, Huang’s group identified a new secretory protein, M28 aminopeptidase (M28AP). The molecular functions and characteristics of M28AP protein were studied by using anti-M28AP antibody and M28AP mutant produced by CRISPR/Cas9 system. The results showed that M28AP was involved in the degradation of human complement, such as C3b and iC3b, suggesting it played a vital role in pathology (Huang et al., 2019). The study also found that *Acanthamoeba* is usually associated with biofilms distributed throughout the environment. It has been confirmed that biofilms can promote protozoan infection (Schaumberg et al., 1998). The biofilm provides a protective niche for *Acanthamoeba*, which is conducive to immune escape, thereby enhancing invasiveness (Marciano-Cabral and Cabral, 2003; Lorenzo-Morales et al., 2015; Hasby Saad and Khalil, 2018) and thought to even provide nutrition for *Acanthamoeba* (Khan, 2006).

## 4. Conclusion

In conclusion, *Acanthamoeba*, is an opportunistic pathogenic protozoa widely existing in nature that can cause various diseases such as AK, GAE, and CA, as well as lung infections. Although its incidence is currently low, resulting infection is serious and treatment options are lacking due to understudied complex pathogenic mechanisms. An in-depth understanding of the biological characteristics and pathogenicity of *Acanthamoeba* can provide help for clinical diagnosis, effective treatment, and control of *Acanthamoeba* infection to provide a theoretical basis for the development of new drugs and vaccines against *Acanthamoeba*.

Although the widely used classification method based on 18S rRNA sequence is not as reliable as full-sequence gene analysis, it provides a rapid, simple and relatively accurate method for the study of genetic diversity of *Acanthamoeba*, which can assist in the differential diagnosis of pathogens in *Acanthamoeba* spp. The identification of *Acanthamoeba* from other cyst forming protozoa can be performed by a method based on the coupling of cellulose binding protein to fluorescent dyes. The main component of cyst wall is chitin, however, *Acanthamoeba* is an exception since its cyst wall contains cellulose (Garajová et al., 2019). Specific cytochemical differentiation between cellulose and chitin by microscopy has not been possible due to the similarity of the constituent β-1, 4-linked hexose backbones of these molecules. Therefore, it is necessary to develop new methods to distinguish cellulose from chitin in order to identify the source of infection. Derda et al. (2009) developed a novel immunocytochemical method for identification of *Acanthamoeba* spp. Cellulose-binding protein consisting of two cellulose-binding domains (CBDs) from Trichoderma reesei cellulase coupled to fluorescent dyes. No staining reaction was observed with the chitin-containing cyst walls of *Giardia intestinalis*, *Entamoeba dispar*, or *Pneumocystis carinii*. Thus, the recombinant CBD can be used as a marker to distinguish between cellulose and chitin (Derda et al., 2009). However, in later studies, cellulose was also identified as a cyst wall component of *Balamuthia mandrillaris*, which is closely related to amoeba (Siddiqui et al., 2009). Even so, it still helps to narrow the scope of differential diagnosis.

Current studies have found that *Acanthamoeba* produces a variety of proteases in the process of infecting the host, which may be an avenue for therapy. Still, the exact molecular mechanism is unclear. Future research should further explore the feasibility of protease inhibitors as therapeutic treatments. The cyst transformation function of *Acanthamoeba* increases the pathogenicity and increases the difficulty of clinical treatment. Therefore, the screening of *Acanthamoeba* cyst formation inhibitors may also prove fruitful for clinical treatment of *Acanthamoeba*. Finally, the unique morphological structure of cysts gives *Acanthamoeba* a remarkable ability to adapt to the environment and may provide another target for clinical treatment.

## Author contributions

YW and ML wrote the original draft. YZ, XJ, LW, and LJ contributed to collecting the data. YW and LZJ contributed to curating the data into figures. RF contributed to revising the manuscript. ML conceived the idea and supervised the work. All authors read and agreed to the final version of the manuscript.
